# Bacterial Infection of an Alveolar Echinococcus Cyst from *C. perfringens* Septicemia: A Case Report and Review of the Literature

**DOI:** 10.3390/medicina59101828

**Published:** 2023-10-13

**Authors:** Jonas Buttenschoen, Vlad Pavel, Alexander Mehrl, Bernhard Michels, Sheila Albaladejo Fuertes, Bettina Seydel, Sophie Schlosser-Hupf, Martina Müller, Stephan Schmid

**Affiliations:** Department of Internal Medicine I, Gastroenterology, Hepatology, Endocrinology, Rheumatology, and Infectious Diseases, University Hospital Regensburg, 93053 Regensburg, Germany; jonas.buttenschoen@ukr.de (J.B.); vlad.pavel@ukr.de (V.P.); alexander.mehrl@ukr.de (A.M.); bernhard.michels@ukr.de (B.M.); sheila.albaladejo-fuertes@klinik.uni-regensburg.de (S.A.F.); bettina.seydel@ukr.de (B.S.); sophie.schlosser-hupf@ukr.de (S.S.-H.); martina.mueller-schilling@ukr.de (M.M.)

**Keywords:** echinococcosis, Clostridiacae, bacterial superinfection, complex liver cysts, liver abscess

## Abstract

*Background and Objectives*: Alveolar echinococcosis (AE) is a highly variable disease able to present as structurally diverse cysts in different organs based on the host’s immunological state as well as the time between diagnosis and the primary infection. Bacterial superinfections, especially with anaerobic pathogens from the Clostridiaceae genus, can further alter the radiological findings due to pneumobilia, newly formed abscess formations, and inflammatory changes. *Materials and Methods*: We present a case of a 71-year-old Caucasian male admitted to our intensive care unit with septic shock, pneumobilia, and a complex cyst of the liver with calcification, as shown by an initial CT. Because of the septic shock, the patient was started on broad-band antibiotics. Clostridiaceae infection was considered an important differential diagnosis due to the presence of pneumobilia observed in the initial CT, without a history of previous endoscopy. Furthermore, serology for echinococcus was positive, and blood cultures showed growth of *C. perfringens*. Therefore, the patient was additionally treated with albendazole. After recovery, further staging was conducted, showing complete remission of the cyst and a left-over lesion classified as Alveolar Echinococcosis Ulm Classification (AEUC) V. In summary, the patient had a pre-existing, controlled AE infection that became superinfected with *C. perfringens*, likely attributable to the anaerobic necrotic tissue, leading to septicemia. *Results*: The anaerobic tissue within the AE cyst provided an ideal medium for *C. perfringens* to replicate, leading to cyst infection, which subsequently caused septic shock and pneumobilia. The initial findings from CT and MRI were confounded by the superinfection, demonstrating the diagnostic challenges of AE, especially when presenting with complications. *Conclusions*: Diagnosing AE remains a demanding task, even with the excellent tools available through serology, coupled with CT, FDG-PET-CT, and MRI. Notably, older superinfected cysts can pose difficulties when integrated into the appropriate diagnostic context. Prompt diagnosis is critical for the accurate treatment of echinococcosis and its complications, such as bacterial superinfections. From a clinical perspective, septicemia from Clostridiaceae and infections with *C. perfringens*—pathogens capable of inducing pneumobilia—should be regarded as significant differential diagnoses for pneumobilia in the absence of a recent history of endoscopy.

## 1. Background

### 1.1. Clostridiacae spp. Septicemia

*Clostridium perfringens* is a rod-shaped, Gram-positive, spore-forming, obligatory anaerobic bacillus ubiquitous to nature and the human gastrointestinal tract [1]. It is most known for food poisoning but also for more severe conditions such as necrotizing myonecrosis. It is classified into seven different toxin types (A–G) based on the different toxins it can produce [1]. Fulminant sepsis with *C. perfringens* is rare but lethal, especially when presenting hemolysis with reported mortality rates between 70 and 100% [2]. Due to its ubiquitous nature, minor injuries while gardening or other entry sites like surgery are typical. However, non-traumatic septicemia has been reported as well [3,4,5]. Clinically, sepsis with *C. perfringens* features a reduced GCS, epigastric pain, vomiting and nausea, and other shock-associated symptoms [5]. Antibiotic treatment includes penicillin, clindamycin, metronidazole, and rifampicin. In case of gas gangrene disease, surgical resection is necessary. Other *Clostridiacae* species have also been isolated in septic patients; however, *C. perfringens* is the most commonly isolated [2].

### 1.2. Hydatid Diseases

Hydatid disease, albeit a radiological diagnosis, is a group of diseases caused by the *Echinococcus*, *Versteria*, *Taenia*, and *Hydatigera* genera [6]. Of particular interest in central Europe and China are echinococci, especially the endemic *E. multilocularis*, which also plays a role as an emerging infectious disease in North America [7]. *E. cysticus* is found worldwide while being endemic in the poorer countries of the world [8]. Humans play the role of an accidental host in the life cycle, becoming infected most likely through contact with animals, especially dogs in rural areas [9]. Once infected, the echinococcus larvae develop mostly in the host liver but can also develop in the spleen, brain, or lungs, causing cystic-, cyst-like, or tumor-like lesions. The leading site of disease manifestation is the liver, which is affected in 98% of the cases. The best treatment option, if caught early on, is surgery, if possible, or otherwise long-term chemotherapy with benzimidazoles, preferably albendazole [10,11]. For cystic echinococcosis, the WHO-Informal Working Groups on Echinococcosis (IWGE) ultrasound classification is typically used.

Due to its tumor-like invasive growth, the alveolar echinococcosis infection remains asymptomatic until the cysts rupture or other structures are invaded. As *E. multilocularis* is not able to mature to its metacestode form and develop a laminated layer like *E. cysticus*, it grows invasive like a tumor trying to find tissues more suitable for itself, with cyst formation being a later occurrence mostly due to necrosis [12]. Alveolar echinococcosis can be classified by computed tomography (CT) scan using the “Alveolar Echinococcosis Ulm Classification” (AEUC), which also gives an idea of how advanced the disease is, i.e., AEUC I is an initial stage, while AEUC II–IV are progressive stages, and AEUC V is a regressive stage [13]. The modified magnetic resonance imaging (MRI) Kodama-XUUB classification can further be used to classify the alveolar echinococcus lesions with regard to their clinical stage by using T2-weighted MRI images [14]. Additionally, ^18^F-FDG-PET-CT can be used to demonstrate metabolic activity as a strong link has been demonstrated between hypermetabolic activity and parasite viability [13,14]. Particularly, delayed acquisition of the ^18^F-FDG-PET (one hour and three hours) can enhance the sensitivity of parasitic metabolic activity [15]. Diagnosis is challenging; therefore, delayed diagnosis of alveolar echinococcosis may lead to postponed initiation of treatment, particularly when the disease is too advanced for resection. One study has shown that 32.5% of cases later revealed to be cystic echinococcosis were initially misdiagnosed [16]. Typical misdiagnoses include cholangiocellular carcinoma, hepatocellular cancer, and atypical and typical liver cysts. Using a multi-modal approach including ultrasound, CT (AEUC I–V classification), MRI (T2w-based Kodama-XUUB classification), and serology, the differential diagnosis of echinococcosis, if considered, can be established to a satisfactory degree.

### 1.3. Superinfections of Hydatid Diseases

Superinfections of hydatid disease are rare. These infections can be blood-borne, retrograde via fistulas, or idiopathic [17]. In a retrospective study, around 7.3% of *E. granulosus* cysts were superinfected upon diagnosis [18]. The most common pathogens found were *E. coli*, *P. aeruginosa*, and *Enterobacter* spp. 83% of cases were initially suggested to be hydatid-related according to CT/MRI findings [18]. Treatment of superinfected cysts should mirror the treatment strategy of abscesses, consisting of drainage and antibiotics, in addition to benzimidazoles [12]. Data on superinfections regarding *E. multilocularis* is rare but has also been reported. The findings in MRI and/or CT when superinfected are not necessarily typical of echinococcus cysts according to the AEUC classification, as shown in one case report [19]. It is also unclear whether superinfections alter the course of hydatid disease or the biology of the cysts and parasites. Based on other experiments involving parasite and bacterial antagonism, inhibition of either is plausible [20].

### 1.4. Serological Tests of Echinococcus Infections

Serology is a valuable diagnostic tool in the assessment of echinococcus infections. A variety of ELISAs are commercially available, including crude antigen, Em2plus, Em10, and Em18 ELISAs, immunoblots, as well as indirect heme-agglutination tests (IHAT). The crude antigen ELISA, as well as the *E. granulosus* IHAT, are good screening tools with high sensitivities up to 80–90% depending on the WHO-IWGE (Informal Working Groups on Echinococcosis) stage [21,22]. The Em10 ELISA is often used to confirm a positive case, with a sensitivity of 93.2% and a specificity of 96.8% for AE and a sensitivity of 89,1% and a specificity of 98.6% for cystic echinococcosis (CE) [23]. There exists a clear connection between cyst stage and serological test results, which therefore leads to the recommendation to consider cyst staging when performing serological tests, at least for CE [22]. Neither antigen-B (AgB)-ELISA nor Em18/Em10 ELISA are specific to AE or CE, leading to cross-reactivity in the later stages of the disease [24].

Many studies have been performed to assess the kinetics of the serologies to determine a good parameter for successful treatment and relapses. Initial PNM staging correlated to the height of the initial serology levels [25]. The highest levels of antibodies are present in patients with unresectable disease [25]. The crude antigen ELISA, once positive, has not been shown to drop below the cut-off values after treatment and should, therefore, not be used for monitoring of the treatment response but for screening purposes [9]. Patients undergoing resection showed an initial increase in antibody titers after surgery [25]. After resection, titers of, especially, the Em2plus-assay drop off, demonstrating its use as a good indicator of successful resection [25].

After chemotherapy with benzimidazoles, IHAT antibodies decrease, while AgB-IgG remains for years after therapy, even after the cyst has receded in imaging [26]. Em2plus, Em10, and Em18 antibodies can decrease below cut-off after treatment, typically after 6–72 months after treatment [25]. The Em18-assay has been shown to best mirror clinical PNM staging and is, therefore, a valuable tool for screening both remission as well as relapsing AE [27,28]. Higher AgB-IgG has been shown in patients with progressive disease than with stable or cured, having potential as an indicator of prognosis [26]. In cases where the patient is immunosuppressed, the serology can be negative and should not be used to rule out the differential diagnosis of echinococcus infection [9].

## 2. Case Presentation

A 71-year-old Caucasian man presented at an emergency room in Bavaria, Germany, with a GCS of 5, and requiring oxygen supplementation via a mask. Furthermore, an elevated heart rate (ca. 120 bpm) and an elevated respiratory rate were noted (23/min). In the patient’s history, only elevated blood pressure treated with candesartan and hydrochlorothiazide was documented. The laboratory work-up showed elevated kidney markers (creatinine: 1.82 mg/dL and urea: 176 mg/dL), elevated liver enzymes (AST: 209 U/L, ALT 135 U/L, bilirubin 22.1 mg/dL), reduced coagulation (INR 1.29, Quick 65%, platelets 71/nL), increased LDH (915 U/L), anemia (hemoglobin 11.1 g/dL) without signs for hemolysis, as well as elevated inflammation markers (CRP 386 mg/L, PCT 19.40 ng/mL, leukocytes 21.05/nL).

**Figure 1:**
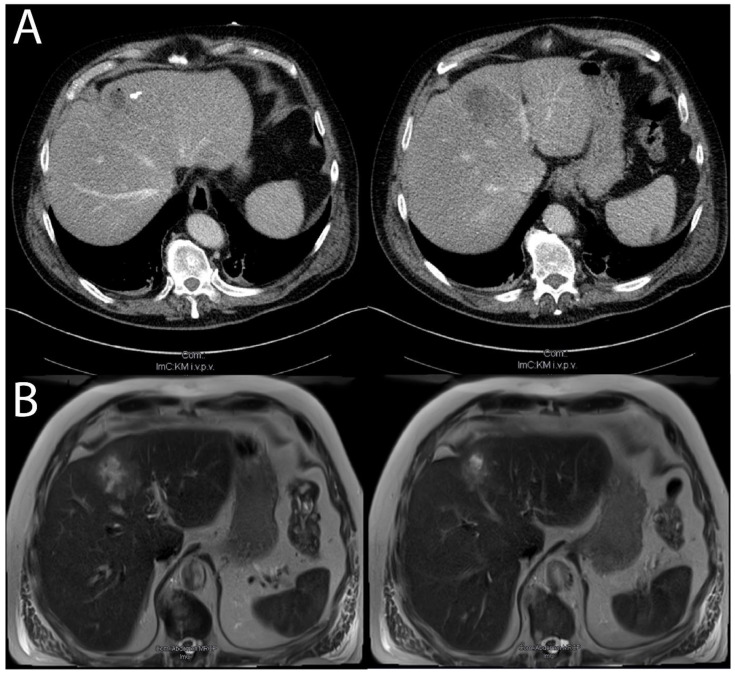
Superinfected echinococcus cyst shown in (**A**): CT with a contrast-enhancing agent (Accupaque) during the septic stage of disease showing a focal hypodense lesion with central air inclusions in segment IVa/IVb with calcifications on its edge (captured with Siemens SOMATOM Definition Flash, Siemens, Munich, Germany). (**B**): MRI, T2-weighted, of the lesion six days later after the patient had stabilized, showing a multi-chambered cyst with air inclusions and microcysts in segment IVa/IVb (captured with Siemens MAGNETOM Sola 1.5T, Siemens, Munich, Germany).

An initial CT scan shown in Figure 1A revealed pneumobilia without a previous history of endoscopy and a lesion of the liver in segment IVa fitting AEUC III with focal calcification or AEUC V with an abscess formation in the residual cyst. In contrast-enhanced ultrasound, the lesion was shown not to have any uptake of the contrast-enhancing agent. A serology for crude-antigen echinococcus was reported positive without hypereosinophilia. Due to suspected echinococcosis, the patient was started on albendazole. The pneumobilia led to the consideration of Clostridiacae infection as a differential diagnosis. An initial empiric antibiosis with penicillin, metronidazole, clindamycin, and meropenem was started. Anaerobic blood cultures revealed growth of *Clostridium perfringens* and *E. coli*. Accordingly, the antibiotic regime was de-escalated to meropenem, metronidazole, and penicillin, under which the patient quickly stabilized, and a significant decrease in inflammation markers was noted. After five days of intensive care treatment the patient was able to be transferred to standard care. After recovering from sepsis, the patient revealed extensive gloveless gardening and minor hand injuries in recent days, likely providing an entry point for the bacteria.

**Figure 2:**
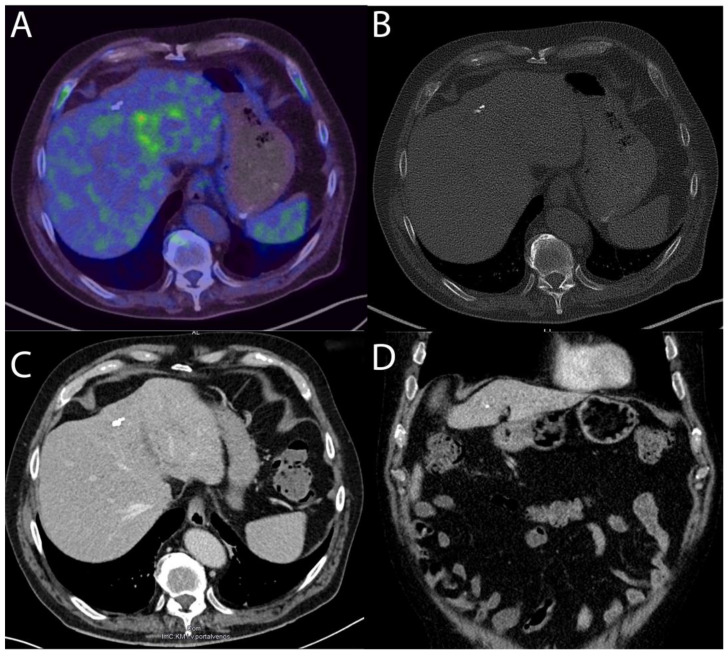
Follow-up imaging for staging. (**A**): PET-CT with delayed acquisition after 3 h showing a polyform ^18^F-FDG enhancement in projection onto the irregular hypodense lesions with an SUV of max. 5.45 (captured with Siemens Biograph 40/64 True Point, Siemens, Munich, Germany). (**B**): Native CT performed as part of the FDG-PET-CT showing slight micro and macro calcifications. (**C**,**D**): Contrast-enhanced CT using Accupaque, captured in axial and frontal planes with a Siemens SOMATOM Definition Flash, demonstrating persistent calcifications and regression of the focal hypodense lesion.

The patient fully recovered after ten more days in standard care and was discharged without clinical symptoms. Creatinine, urea, bilirubin, liver enzymes, and inflammation markers normalized by discharge. An ultrasound-guided fine-needle aspiration to confirm the AE was not performed as no proper puncture window for a biopsy was available. A follow-up appointment after six weeks, during which the serology was repeated, showed positive antibodies against AgB and Em10, while the crude-antigen Ig had turned negative. An MRI of the liver shown in Figure 1B revealed a lesion most likely classified as Kodama-XUUB IIIa, demonstrating an older, inactive cyst. As shown in Figure 2A, ^18^F-FDG-PET-CT showed no metabolic activity of the lesion. The CT performed as part of the ^18^F-FDG-PET-CT as well as a contrast-enhanced CT, as shown in Figure 2B,D, demonstrated a liver lesion that could be classified as AEUC V, thus demonstrating a remarkable recession of the lesion. During a further follow-up visit with ultrasound, the lesion demonstrated no further changes.

## 3. Discussion

This case demonstrates that pneumobilia without a recent history of endoscopy, especially ERCP, should be regarded as a caution sign that cannot be ignored. Especially combined with further clinical presentation of septicemia or septic shock, an infection, including abscess or superinfection of a liver cyst, must be considered. Due to their ubiquitous nature, Clostridiacae, including *C. perfringens*, should be considered as a differential diagnosis and should be included in the empiric treatment.

This clinically interesting case involves an asymptomatic and previously controlled old *E. multilocularis* cyst, which became superinfected, leading to severe septicemia with septic shock. The initial diagnosis was established with CT and MRI imaging (AEUC III, Kodama-XUUB IIIa) in combination with a positive-crude-antigen ELISA. As the crude-antigen ELISA is cross-reactive for both *E. cysticus* and *E. multilocularis*, specific ELISAs were performed but were initially negative. One issue with this is that this serology was taken during the septic state representing a kind of immune-suppressed state, possibly skewing the serologic results. Due to clinical suspicion, the patient was started on albendazole based on a high chance of hydatid disease, considering the imaging results. At the time of follow-up, six weeks later, the AgB-IgG and Em10 ELISA turned positive, while the crude-antigen ELISA stayed negative. While it is unusual for the crude antigen ELISA to turn negative even after a long treatment period, it is reasonable to assume that the superinfected cyst is an older cyst, or abscess, which the immune system did control until the superinfection occurred [12,25]. It is also possible that the superinfection with *C. perfringens* caused the release of antigens from the cyst, causing the immune system to be re-introduced to the parasite fragments, consequently leading to rapid antibody production. In the follow-up imaging, the lesion showed itself to be regressive, possibly as the superinfection with *C. perfringens* broke the established laminated layer and fibrin layers allowing for the tissue to regenerate and clear the necrosis.

While echinococcosis diagnostics have improved to satisfactory degrees in the last decade, including excellent results from the serologies, this case demonstrates that AE remains a complex diagnosis with many factors that need to be considered, including the age of the cysts, the degree to which the immune system is controlling the infection, as well as complications such as superinfections of older or necrotic cysts.

## 4. Conclusions

This clinical case presentation demonstrates that alveolar echinococcosis should be treated in a multi-disciplinary specialized center. Alveolar echinococcosis should always be considered as a differential of complicated or uncommon cysts, while Clostridiacae infections may always be considered as a relevant differential diagnosis of pneumobilia.

## Data Availability

The datasets generated and/or analyzed during the current study are not publicly available due to data privacy but are available from the corresponding author on request.

## Abbreviations

AE	Alveolar echinococcosis
AEUC	Alveolar Echinococcus Ulm Classification
ALT	Alanine aminotransferase
AST	Aspartate aminotransferase
CE	Cystic Echinococcosis
CRP	C-reactive peptide
CT	Computed tomography
ELISA	Enzyme-linked immunosorbent assay
GCS	Glasgow Coma Scale
IHAT	Indirect Hemagglutination test
INR	International normalized ratio
LDH	Lactate dehydrogenase
MRI	Magnet resonance imaging
PCT	Procalcitonin
WHO-IWGE	World Health Organisation-Informal working groups on Echinococcus
